# Proxy evidence for state-dependence of climate sensitivity in the Eocene greenhouse

**DOI:** 10.1038/s41467-020-17887-x

**Published:** 2020-09-07

**Authors:** E. Anagnostou, E. H. John, T. L. Babila, P. F. Sexton, A. Ridgwell, D. J. Lunt, P. N. Pearson, T. B. Chalk, R. D. Pancost, G. L. Foster

**Affiliations:** 1 GEOMAR Helmholtz-Zentrum für Ozeanforschung Kiel, Wischhofstrasse 1-3, 24148 Kiel, Germany; 2 School of Ocean and Earth Science, National Oceanography Centre Southampton, University of Southampton Waterfront Campus, Southampton, SO14 3ZH UK; 3 School of Earth and Environmental Sciences, Cardiff University, Park Place, Cardiff, CF10 3AT UK; 4 School of Environment, Earth and Ecosystem Sciences, The Open University, Milton Keynes, MK7 6AA UK; 5 Department of Earth Sciences, University of California, Riverside, CA 92521 USA; 6 School of Geographical Sciences, University of Bristol, University Rd, Bristol, BS8 1SS UK; 7 Organic Geochemistry Unit, School of Chemistry and School of Earth Sciences, Cabot Institute for the Environment, University of Bristol, Queens Rd, Bristol, BS8 1UJ UK

**Keywords:** Climate sciences, Palaeoceanography, Palaeoclimate

## Abstract

Despite recent advances, the link between the evolution of atmospheric CO_2_ and climate during the Eocene greenhouse remains uncertain. In particular, modelling studies suggest that in order to achieve the global warmth that characterised the early Eocene, warmer climates must be more sensitive to CO_2_ forcing than colder climates. Here, we test this assertion in the geological record by combining a new high-resolution boron isotope-based CO_2_ record with novel estimates of Global Mean Temperature. We find that Equilibrium Climate Sensitivity (ECS) was indeed higher during the warmest intervals of the Eocene, agreeing well with recent model simulations, and declined through the Eocene as global climate cooled. These observations indicate that the canonical IPCC range of ECS (1.5 to 4.5 °C per doubling) is unlikely to be appropriate for high-CO_2_ warm climates of the past, and the state dependency of ECS may play an increasingly important role in determining the state of future climate as the Earth continues to warm.

## Abstract (web summary)

The relationship between atmospheric CO_2_ and climate during the Eocene greenhouse remains uncertain. Here authors show that Eocene CO_2_ and climate sensitivity was high during the warmest intervals and declined as global climate cooled, with implications for the Earth’s future warming climate.

## Introduction

The Eocene Epoch is the most recent greenhouse period in Earth’s history. Atmospheric carbon dioxide (CO_2_) and temperature peaked in the early Eocene, and both declined towards the late Eocene, ultimately leading to an icehouse state at the Eocene-Oligocene Transition (e.g. refs. ^1–5^). However, to better constrain the potential mechanisms driving the early Eocene warmth and the subsequent cooling, high-resolution records of CO_2_ and temperature are required. While obtaining continuous marine records of temperature through this interval has been an ongoing effort (e.g. refs. ^1,2^), similar records for CO_2,_ as compiled in ref. ^3^, are fragmented and of low temporal resolution with large uncertainties, and thus remain insufficient to fully characterise the climate dynamics of the Eocene.

Of particular importance in this regard are several recent modelling studies that have highlighted the possible existence of a state-dependency of climate sensitivity. That is, the magnitude of global mean temperature change following a doubling of atmospheric CO_2_ is higher in warm climates than in cooler periods, including the modern climate system (e.g. refs. ^1,6–8^). In the Eocene, this is thought to result from non-linearities in the albedo response related to cloud feedbacks rather than snow and ice feedbacks^6–8^. These feedbacks are further modified by changing palaeogeography, potentially linked to ocean area and deep water formation^8^. Given the major implications such a state dependency may have on the amount of warming by 2100 and beyond under high-emission scenarios (e.g. RCP8.5), there is a pressing need for improved constraints on the nature and evolution of climate sensitivity in different climate states.

**Fig. 1:**
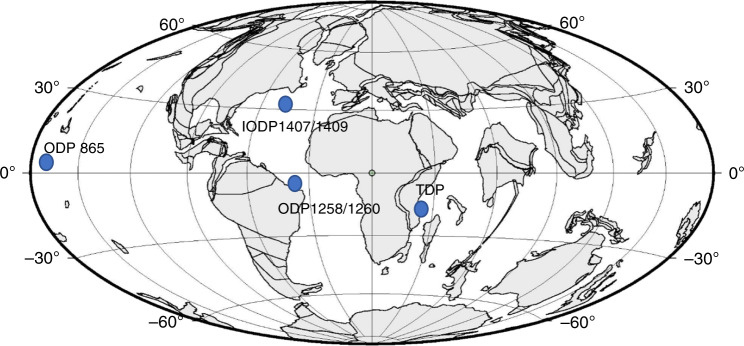
Paleo-location of sites used in this study. Base map generated from www.odsn.de for the early Eocene.

In order to achieve this, we generate a new CO_2_ record, spanning the Eocene Epoch with an average sampling resolution of 1 sample per 0.25 million years (Myr), using boron isotopes (δ^11^B) in planktonic foraminifera from five pelagic sites located in the Atlantic and Pacific: International Ocean Discovery Program (IODP) Sites 1407 and 1409, Newfoundland margin; Ocean Drilling Program (ODP) Sites 1258 and 1260, Demerara Rise, and ODP Site 865, Allison Guyot, (Fig. 1). This record, coupled to existing δ^11^B-CO_2_ reconstructions^4,5,9–11^ and novel Global Mean Temperature (GMT) estimates, is used to provide proxy evidence of the state dependency in climate sensitivity, with higher sensitivity during the warm period of the early Eocene, and lower towards the transition to the colder, late Eocene.

## Results and discussion

### Reconstructions of seawater pH

We followed established methods to calculate seawater pH and CO_2_ from foraminiferal δ^11^B measurements^4,12–14^ (“Methods”). We employ the δ^11^B proxy on mixed-layer species of planktonic foraminifera in all core sites to first reconstruct surface ocean pH. The majority of Paleogene foraminiferal species selected for this study were previously identified to reflect surface mixed layer conditions^4,10^, and are likely characterized by a reduction in the degree of pH modification in the micro-environment surrounding the foraminifera by physiological processes compared to observations in modern foraminifera^4,14^. When thermocline dwelling species were used, or additional species not previously analysed, we ensured that our new analyses of δ^11^B overlapped with previously studied mixed-layer planktonic foraminiferal species (“Methods” and Supplementary Data 1) in order to constrain site-specific intra-species offsets and thus provide consistency and confidence in the derived mixed-layer pH (as in ref. ^4^). Seawater temperatures for the calculation of carbonate system parameters from δ^11^B were estimated using foraminiferal Magnesium/Calcium (Mg/Ca) ratios determined on an aliquot of the same solution used for δ^11^B analyses, assuming Eocene seawater Mg/Ca of 2.2 ± 0.1 mol/mol^2,4^ and the seawater adjusted Mg/Ca thermometer^15^.

### Reconstructions of atmospheric CO_2_

The derived surface seawater pH estimates from foraminiferal δ^11^B were combined with the latitude-specific estimates of calcite saturation in surface waters (from cGENIE^4^), which we assume remains within a range of ±1, thereby accounting for uncertainty in both absolute value and any short-term variability^16^. Full error propagation was carried out using a Monte Carlo approach as described in ref. ^4^. The CO_2_ record was then smoothed using varying span LOESS curve with the degree of smoothing optimised using generalised cross validation (Michael Friendly: https://tolstoy.newcastle.edu.au/R/help/05/11/15899.html). The 95% confidence intervals were then estimated from smoothing the residuals between the LOESS curve and the CO_2_ data.

### Eocene time-series of δ^11^B-derived pH and CO_2_

**Fig. 2:**
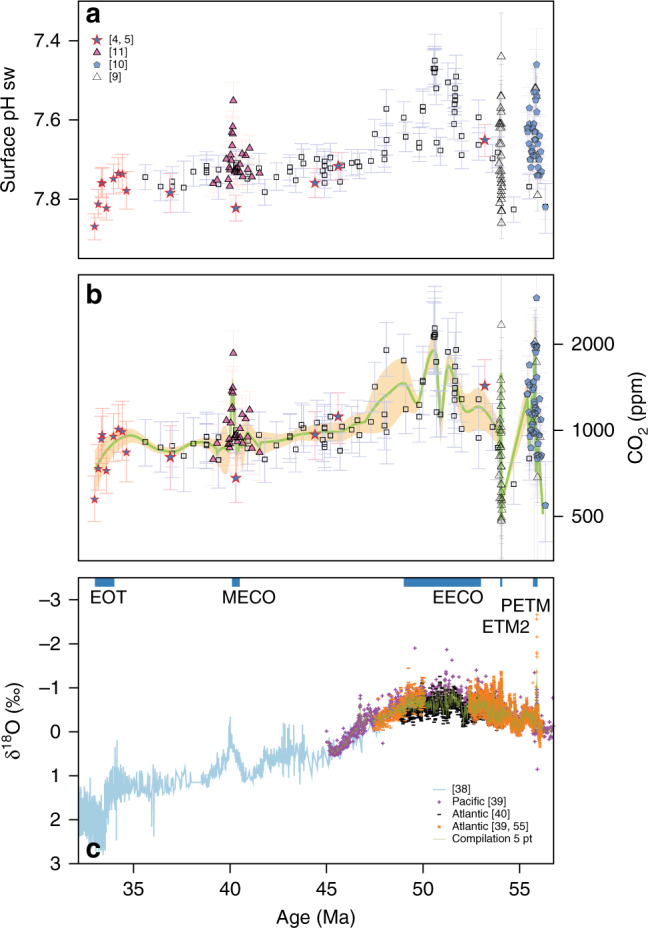
Compilation of δ^11^B and δ^18^O derived records for the Eocene. **a** Seawater pH from the new δ^11^B data presented here (black squares) and compiled from the literature (see panel for appropriate references), all listed in Supplementary Data 1 and 3, **b** calculated atmospheric CO_2_ from the data shown in **a**, the LOESS fit (green line) and 95% confidence (orange shading) (see “Methods” for details), **c** δ^18^O from benthic foraminifera are based on compilations (see Methods for individual references). Error bars in **a**, **b** are 95% confidence. Intervals of time referred to in the text are shown as blue bars in **c**, labelled with appropriate acronym.

Our new continuous and high-resolution record of δ^11^B-derived pH and CO_2_ (Fig. 2, Supplementary Fig. 1) overlaps with existing low-resolution δ^11^B -based records from Tanzania^4,5^, and records from the Middle Eocene Climatic Optimum (MECO; ~40.1–40.5 Ma)^11^, Eocene Thermal Maximum 2 (ETM2; 54.1 Ma)^9^, and the Paleocene-Eocene Thermal Maximum (PETM; ~56 Ma)^9,10^ (all re-calculated for consistency, see “Methods” and Supplementary Data 3), and demonstrates the validity of our multi-species treatment of δ^11^B in deriving mixed-layer pH and CO_2_ concentrations. This continuous view of the evolution of CO_2_ confirms that the highest CO_2_ levels, outside of the short-lived increase in CO_2_ at the PETM^9,10,17^, occurred during the Early Eocene Climatic Optimum (EECO; 49–53 Ma^18^). Pre-PETM CO_2_ was ca. 900 ± 100 ppm (±2 se, *n* = 14)^9,10^. For the EECO and the PETM^9,10^, the average CO_2_, calculated using the average δ^11^B and Mg/Ca-temperature estimates in each interval is 1470 (+360/−300) ppm (2 s.d.) and 1790 (+560/−380) ppm respectively (or 1980 (+510/−440) ppm and 2470 (+690/−540) (2 s.d.) if the *Trilobatus sacculifer* calibration of ref. ^19^ is used, as described in the “Methods”). Atmospheric CO_2_ began to decline from a maximum at ca. 49 Ma, reaching a minimum immediately prior to the MECO^4,11^ where it increased to an average of 1240 (+250/−210) ppm (or 1490 (+290/−240) ppm using the *T. sacculifer* calibration)^18^. Following the MECO, CO_2_ levels remain largely stable at 900 ± 130 ppm (2 s.d.) until the Eocene-Oligocene transition (EOT; 33.5–34 Ma), when they eventually decline below 700 ppm^4,5^.

### Atmospheric CO_2_, volcanism and silicate weathering

**Fig. 3:**
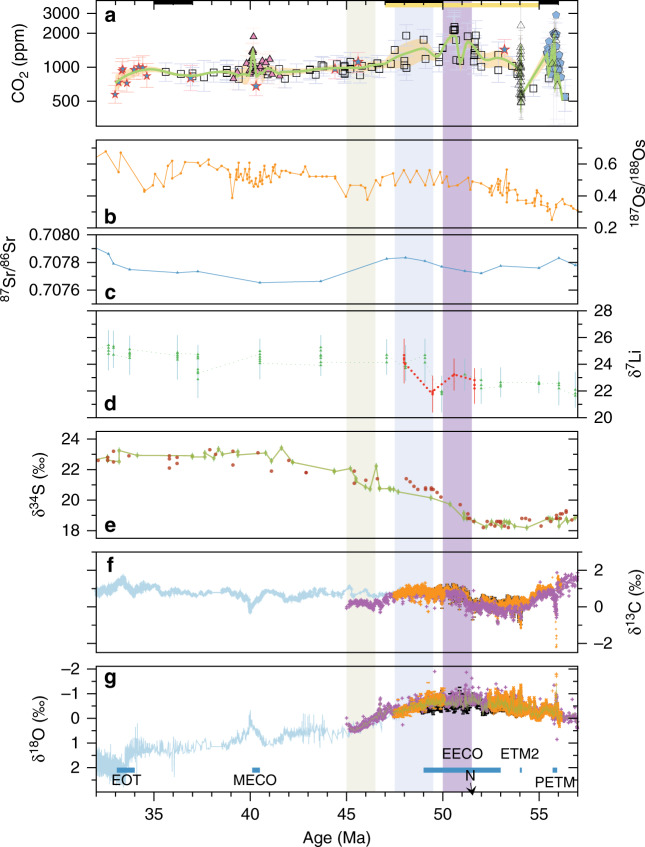
Early Eocene weathering, organic carbon burial and circulation changes. **a** The CO_2_ record as in Fig. 2. **b** The marine ^187^Os/^188^Os compilation from ref. ^27^ **c** the marine ^87^Sr/^86^Sr record from refs. ^30,32,94^. **d** The marine δ^7^Li are from ref. ^33^, with red symbols/line updated to the same age model as for the CO_2_ and δ^18^O data^39^. **e** The marine δ^34^S are from ref. ^51^ (red circles), with the updated age model to GTS2012 (green circles) from ref. ^53^ **f**, **g** Marine carbonate δ^13^C and δ^18^O, color coding refers to the same references as in Fig. 2c. Purple bar envelopes the δ^34^S increase, and “N” indicates the preceding CIE within C23n.2nH1^39^. Light blue bar indicates the timing of temperature and CO_2_ decline after the EECO. Green light bar indicates the timing of potential circulation changes in the early Eocene, as demonstrated in the δ^13^C record of **f**. Overlying solid black and yellow bars represent the timing of volcanism^21–26^.

The most important modulators of the Earth’s carbon cycle, and hence its climate, are thought to be the balance between volcanic CO_2_ output and CO_2_ drawdown through silicate weathering and carbonate burial^20^. However, the relative importance of these processes in determining the evolution of CO_2_ over the last 65 Myr, and hence their role in the evolution of Cenozoic climate, remains uncertain. Our new continuous CO_2_ record allows a re-evaluation of the broad relationship between records of silicate weathering, volcanism and CO_2_ during this interval (Fig. 3).

There is abundant physical evidence for enhanced volcanism during the EECO, potentially driving high levels of CO_2_ during this time (Fig. 3). The central East Greenland volcanic rift margin plutons associated with post-continental break-up were emplaced from 56 to 54 Ma and 50 to 47 Ma^21^, following the flood basalt of North Atlantic Igneous Province emplacement and volcanism associated with the PETM^22^. In addition, in central British Columbia there was extensive magmatism within the Chilcotin Plateau (from 55 to 47 Ma^23^) and the Challis-Kamloops magmatic belt (from 53 to 47  Ma^24,25^). The India-Eurasia collision resulted in the subduction of pelagic carbonates deposited within the Neo-Tethys and of carbonate sediments from the continental margin of the Greater Indian subcontinent, which were most likely recycled as CO_2_ at arc volcanoes from ca. 52.5 to 49 Ma^26^, also coinciding with the elevated CO_2_ during the EECO.

The carbon imprint of silicate weathering on the Eocene carbon cycle remains unconstrained (e.g. refs. ^27,28^) because the available paleoproxies are currently ambiguous and reconstructions tend to be sparse for this time interval^29–32^ (Fig. 3). Only the Li isotope record^33^ reveals a step change in the early Eocene at ca.48 Ma, indicating that a shift toward higher silicate weathering intensity was coincident with our post-EECO CO_2_ decline (Fig. 3d). Such an increase in weathering could be due to the second stage of collision of India with Asia^34,35^, and Patagonian orogenesis^36^ that occurred at around 50-49 Ma. Following the EECO warmth and initial cooling, global cooling and reduced weathering intensity, as implied from Os isotopes (Fig. 3b), may have slowed down the weathering feedback^27,37^ contributing to the nearly stable CO_2_ levels we reconstructed for this time.

### Drivers of the ca. 51 Ma decoupling between δ^13^C and CO_2_

Although the timing of major weathering regime changes and volcanic events coincide with large variations in our CO_2_ curve, there is structure within our record that require the action of additional processes. Previous work indicates that δ^13^C and δ^18^O values are tightly coupled on short-term orbital scales and across hyperthermals such as the PETM (e.g, ref. ^38^); however, they decouple on longer timescales, including in the marked transition from ca. 51 to 51.5 Ma, characterized by a 1–2 ‰ increase in benthic foraminiferal δ^13^C records during the sustained warmth of the EECO (Fig. 3)^39,40^. Our CO_2_ record demonstrates for the first time that this increase in δ^13^C is not associated with a systematic change in CO_2_.

Large scale circulation changes could cause this δ^13^C-CO_2_ decoupling, but they preceded the EECO by ca. 6 My^41^, except the short-lived changes in deep water formation during hyperthermal events, such as the PETM^42^. Additionally, cessation of North Pacific deep-water formation^43^, a more inter-basin thermohaline circulation δ^13^C pattern^44^ (Fig. 3f), and establishment of a proto-Antarctic Circumpolar circulation (proto-ACC) associated with the gradual Drake Passage opening (ref. ^45^) and the Tasman Seaway widening (refs. ^46,47^) followed the EECO CO_2_ and temperature decline (post 47 Ma). Therefore circulation changes are unlikely to have been the main drivers of the δ^13^C and CO_2_ decoupling within the EECO.

Alternatively, this decoupling could arise from multiple changes in carbon sources and sinks. Volcanic carbon emissions could have been associated with a nearly neutral atmospheric δ^13^C signal while still elevating CO_2_ concentrations, such as the case of metamorphic degassing of carbonates, whereas the positive δ^13^C excursion can be explained by enhanced burial of δ^13^C depleted organic carbon^48^. Although the amount of organic carbon burial across the early Eocene remains debated^49,50^, the most striking evidence for organic carbon burial increase is the S isotope record obtained from foraminifera calcite^51^ (Fig. 3e) and sedimentary barite^52,53^, which reveals a sharp increase in δ^34^S of seawater sulfate starting at ca. 52 Ma and is potentially linked to a change in the locus of organic carbon burial and an increase in the burial of organo-sulfides^51,52,54^.

### Global mean temperature and climate sensitivity

**Fig. 4:**
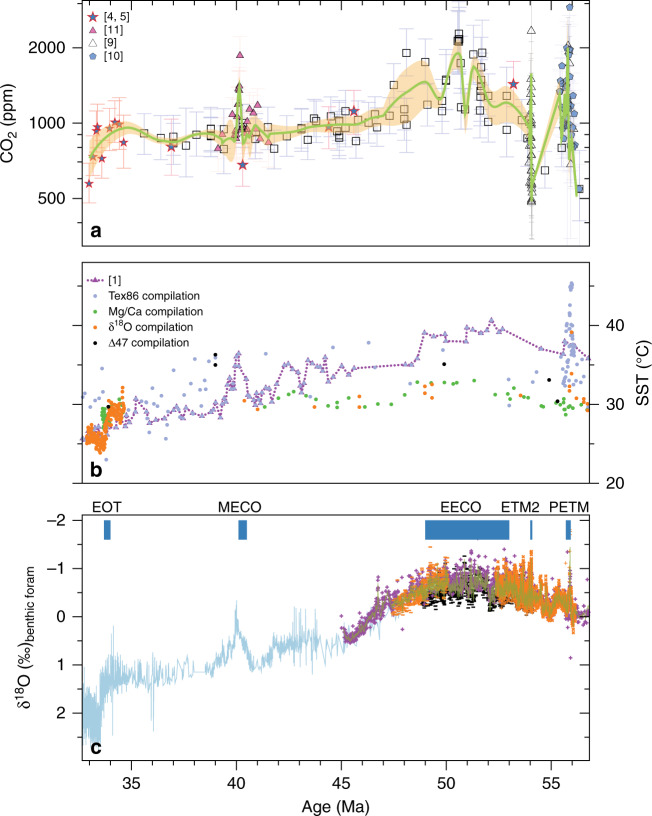
Comparison of the δ^11^Β-derived CO_2_ to temperature records. **a** CO_2_ compilation as in Fig. 2a, **b** Sea surface temperature (SST) records, as compiled in ref. ^1^ (see “Methods” for the list of references used in the compilation). Purple dotted line connects the TEX_86_ record from ODP 959^1^. **c** Benthic foraminifera δ^18^O (related to deep water temperature) as in Fig. 2c.

Regardless of the causes of the evolution of CO_2_ through the early Cenozoic, our new CO_2_ record clearly resembles long-term deep-sea and surface seawater temperature (SST) records as compiled in refs. ^1,2,38–40,55^, (see “Methods”, Fig. 4). To further explore the relationship between CO_2_ and the global mean temperature evolution during the Eocene, we first computed GMT (Methods). However here, rather than using multi-site, non-continuous foraminiferal δ^18^O records^8^, which have also been shown to be impacted by diagenesis^56,57^, we use the continuous TEX_86_-SST record from the equatorial Atlantic (ODP 959)^1^ and the model simulations with the NCAR Community Earth System Model version 1 (CESM 1) in ref. ^1^, which provide a transfer function from SST at ODP 959 to a global mean in four specific time windows (54–49, 48–46, 42–42, 38–35 Ma; Supplementary Fig. 2).

**Fig. 5:**
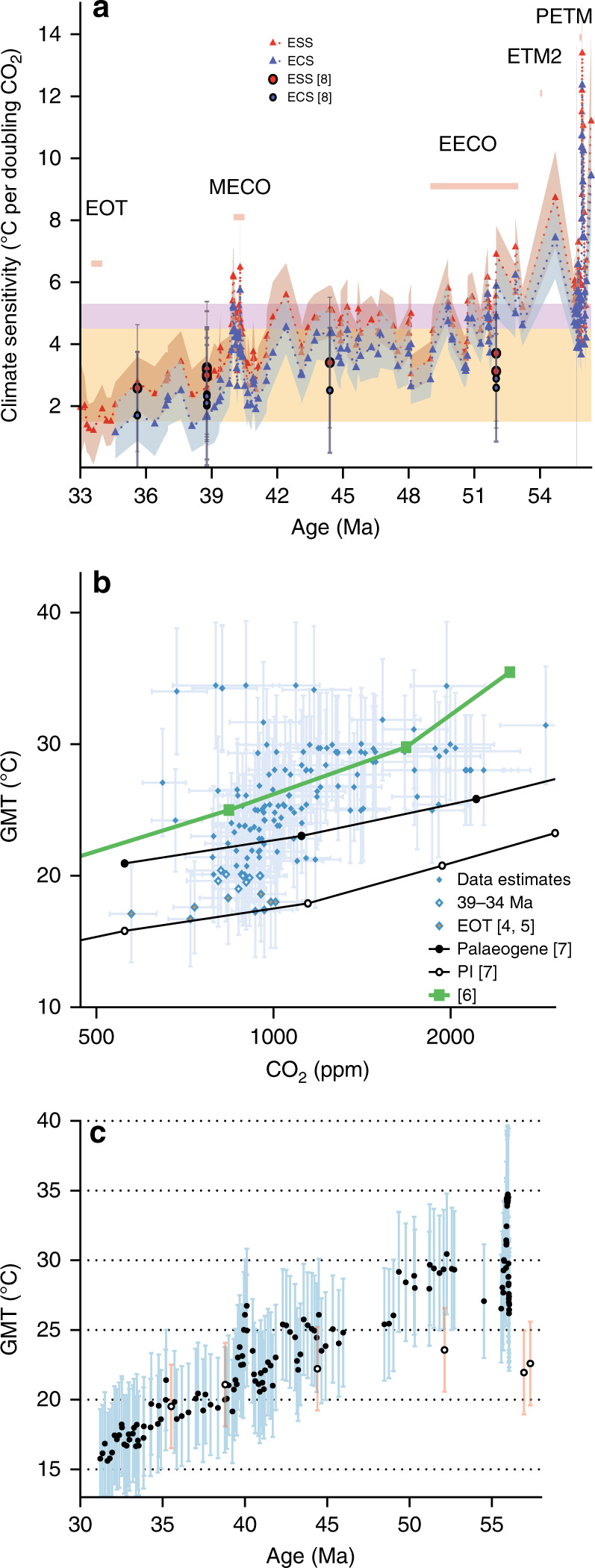
Evolving climate sensitivity for the Eocene. **a** Calculated ESS (red triangles and error envelope), and ECS (blue triangles and error envelope). See text for relevant methodology. Orange area represents the IPCC range in ECS^95^, and the pink highlighted area the updated 20th century ECS with the addition of state-of-the-art cloud physics^96^. Circles represent estimates from ref. ^8^. **b** Data-model inter-comparison, with all diamonds representing data. Open diamonds are the data between 39 and 34 Ma, and orange filled diamonds the EOT. Circles^7^ and squares^6^ are all model derived relationships (PI preindustrial). Uncertainties and error envelopes represent 1 s.d. of Monte Carlo propagated uncertainties. **c** Evolving GMT relationship for the Eocene. GMT is calculated using the BAYSPAR TEX_86_ record from ODP 959. Error bars represent the calibration and analytical uncertainty on TEX_86_. For comparison, the GMT estimates from ref. ^8^ are presented with open symbols and red error bars. NB the elevated ECS early in the PETM and ETM 2 are most likely a consequence of slight age model misalignments, or imply non-CO_2_ forcing early in these events.

The relative change in climate forcing (W m^−2^) within the Eocene attributable to CO_2_ change relative to preindustrial (PI) CO_2_ (278 ppm) (ΔFCO_2_) is calculated using the formulation of ref. ^58^. Earth System Sensitivity (ESS), defined as the mean temperature response to all radiative perturbations^59^, can then be computed from the change in global mean temperature relative to preindustrial (ΔGMT), using the equation:

1 $${\mathrm{ESS = }}\,\Delta {\mathrm{GMT/}}\Delta {\mathrm{FCO}}_2 \times 3.87$$

where the 3.87 W m^−2^ expresses the ESS as the temperature change due to a CO_2_ doubling. However, to isolate the climate change due purely to changes in CO_2_, we must first account for the influence of paleogeography and solar constant on GMT. To do this we subtract a time variant correction following ref. ^8^ estimated to ~0.5 °C in the late Eocene and 1.5 °C in the early Eocene (Supplementary Data 2). Finally, we provide an estimate of Equilibrium Climate Sensitivity (ECS) by accounting for the contribution to Eocene GMT of the changes in the land-ice sheets (equivalent to 1.5 ± 0.5 °C, refs. ^60,61^), a slow-climate feedback not considered in climate models (PALAEOSENS^59^). To calculate ECS in this way we use Eq. (1), but we first subtract from GMT the estimated temperature changes due to solar constant, paleogeography, and ice sheets (Fig. 5a and Supplementary Data 2). Note that we do not provide any corrections for other greenhouse gasses. Finally, to examine the robustness of our findings to our chosen record of GMT we use an independent alternative approach for calculating GMT from ref. ^8^ using foraminiferal δ^18^O (Fig. 5c).

Recently, a number of studies have focused on non-linearities of the climate system during the Eocene, such as those related to changes in paleobathymetry affecting ocean area and deep water formation^8^, and short-wave cloud feedbacks linked to cloud microphysics, amplifying surface warming through changes in clouds^6^. Here we compare our GMT vs. ΔFCO_2_ relationship for the Eocene to climate model derived relationships for different boundary condition and processes (Fig. 5b). Largely independent of the approach used for calculating GMT, the majority of our reconstructions fall within the range of Paleogene simulations in refs. ^6,7^. Our time-evolving record of ECS (and ESS) through the Eocene, even when considering the large uncertainty it inherits from the individual GMT and CO_2_ values used for its calculation, shows that the highest ECS estimates occur consistently during the warm intervals of the Eocene, such as the PETM, ETM2, EECO and MECO, and progressively decline towards the EOT (Fig. 5a).

The declining ECS for the Eocene, and the overlap between our early Eocene climate sensitivity estimates and the model output of ref. ^6^ (Fig. 5b), provide a strong confirmation of state dependency of ECS likely driven by changes in cloud-microphysics^6^. This finding is robust to the uncertainties in final estimates of ECS as it is present in all processing scenarios we consider which largely influence our estimates of absolute ECS, not the pattern of its evolution through time. The decrease in GMT that we observe post 39 Ma (Fig. 5b), however, is not sufficiently described by this early Eocene model, implying that non-CO_2_ boundary conditions may be playing a role in changing climate at this time, such as changes in paleogeography and/or associated changes in ocean circulation, and the presence of ice sheets^8,47,62–65^.

Our new compilation of δ^11^B-CO_2_ from planktonic foraminifera from multiple open ocean sites provides a comprehensive picture of the evolution of CO_2_ through the Eocene, greatly improving on recent CO_2_ compilations (ref. ^3^ and Supplementary Fig. 3) and allowing for the first direct comparison with high-resolution records of climate variability. Our reconstructions, while still underlining the importance of CO_2_ in driving the evolution of Eocene climate, provide evidence of strong non-linearities between climate and CO_2_ forcing, likely related to both cloud feedbacks for the early-mid Eocene, and changing paleogeography and ice sheets for the late Eocene. This reveals climate-state dependent feedbacks and elevated ECS operated during the warmest climates of the last 65 million years.

## Methods

### Site information and age models

Boron isotopes (δ^11^B) from mono-specific samples of planktonic foraminifera were obtained from a number of deep-sea, open-ocean Paleogene-age core locations (Fig. 1). Sites ODP 865 and ODP 1258 and 1260 were positioned in subtropical/tropical paleolatitude and Sites IODP 1407/1409 was likely within temperate latitudes (Fig. 1), and all sites were located within deep-bathyal water depths throughout the Eocene above the calcite compensation depth (CCD)^44,66–68^. Age models for IODP 1407/1409 and ODP 1258/60 were updated to ref. ^39^ timescale.

The age-depth model used for site 865B (Supplementary Table 1 and Supplementary Fig. 4) in this study was based on that from ref. ^69^, with refinements in this study including re-adjustment to the GTS2012^70^ timescale. The model uses a linear fit^71^, but it is solely based on planktonic foraminiferal events (excluding nannofossils), because of suspected winnowing bottom water currents that may have mobilized the fine fraction containing nannofosils, making them suspect. We only used datums for which GTS2012 ages were available and in which we had significant confidence (Supplementary Table 1), such as those without obvious signs of reworking.

At Sites IODP 1407 and 1409 the planktonic foraminifera exhibit *glassy* test textures and appear minimally influenced by post-depositional recrystallization^68^, while at ODP 1258/1260 and ODP 865 the foraminifera specimens are frosty in appearance^57,72^, indicative of partial or complete recrystallization, with the most altered site being ODP 865, without hampering identification of individual species. Nevertheless, it has been shown that at least at ca. 40.3 Ma, ODP 865 δ^11^B of planktonic and benthic foraminifera are indistinguishable from that of glassy, well-preserved foraminifera from the Tanzania Drilling Project (TDP)^73^.

Records of δ^13^C and δ^18^O displayed in Fig. 2 to Fig. 4 were generated from ODP Sites 1258,

1262, 1263, 1265 and 1267 and 1209 in refs. ^40,55,74–81^, on the ref. ^39^ age model, and from Deep Sea Drilling Project and ODP sites in ref. ^38^.

### Sample preparation

Approximately 3 mg of 73 mono-specific planktonic foraminiferal carbonate samples of a narrow size fraction (Supplementary Data 1) were separated from 2 to 10 cm of core material for tandem analyses of boron isotopes and trace element composition. Identification of planktonic foraminifera followed ref. ^57^, and samples were cleaned following established methods^82–84^. Trace element to calcium ratios were determined as in ref. ^84^ and Al/Ca ratios were typically <150 μmol/mol signifying efficient surficial clay removal during the foraminiferal cleaning procedure^84^. For all core sites used in this study, there was no relationship between Al/Ca μmol/mol and foraminiferal δ^11^B measurements, suggesting that any clay remnants did not bias the measured δ^11^B values^10^.

### Mg/Ca analyses, temperature reconstructions

Trace element to calcium analyses were carried out using a Thermo Scientific Element XR sector-field inductively-coupled-plasma mass spectrometer (SF-ICPMS) at the University of Southampton. The long-term precision (2 s.d.) of an in-house carbonate standard was 2% for Mg/Ca (mmol/mol) and Al/Ca (μmol/mol). Seawater temperature was estimated from each sample using foraminiferal Mg/Ca ratio on an aliquot of the same solution used for δ^11^B analyses, assuming Eocene seawater Mg/Ca of 2.2 ± 0.1 mol/mol^2,4^ and Mg/Ca-temperature calibration sensitivity was adjusted based on the seawater Mg/Ca value^15^. The temperature uncertainty is set to a range of ±2 °C and it is fully propagated into our carbonate system estimates (see below).

### Relative δ^11^B offsets

Identification of planktonic foraminiferal depth habitats used in this study are based on relationships between stable isotope foraminiferal geochemistry and ecology in relationship to δ^11^B offsets (e.g., refs. ^4,9,10^, and references therein). Additional foraminifera species used here (*Morozovella aragonensis, Acarinina quetra, A. pentacamerata, M. crater, A. cuneicamerata, A. pseudosubsphaerica*) were cross-calibrated against previously known species (*A. pseudotopilensis, A. praetopilensis, A. soldadoensis, Guembelitrioides nuttalli, Pearsonites broedermanni*) for their δ^11^B behaviour collected from the same time interval and core site^4,5,11,17^ and site-specific species offsets in δ^11^B were not identified. In site ODP 865, the δ^11^B composition of *Turborotalia cerroazulensis*, *T. frontosa*, and *T. ampliapertura* are offset from the mixed layer species *A. rohri*, *A. praetopilensis* and *A. topilensis* by on average 1.02 ± 0.04 (2 s.e., *n* = 3) ‰, confirming previous estimates for *T. ampliapertura*^4^, but showing less of an offset for the species *T. cerroazulensis* compared to TDP^4^, thus we used the site-specific offset here for this species, propagating the uncertainty of this offset correction through the calculations. Sites 1407 and 1409 are dominated by *A. bullbrooki* in the late Eocene which recorded variably lower δ^11^B values than known shallow mixed layer species and so were excluded from the time series compilation. For consistency, we have included previously published δ^11^B records generated from planktonic foraminiferal species we have tested for relative vital effects and interspecies offsets in our timeseries. Therefore, we excluded the *M. velascoensis* record of the PETM^17^, since this species is randomly offset from our tested species *A. soldadoensis* when comparing five samples from site 1209 and at similar ages (Δδ^11^B_*M.velascoensis – A. soldadoensis*_ = 0.8 ± 0.6‰ (2 s.d.)^9^. Also, both *G. index and G. kugleri* records of the MECO^11^ are excluded, because the former showed variable habitat depth and δ^11^B offsets in TDP^4^, and the latter is not sufficiently tested for within site inter species offsets.

### Boron isotope proxy and analyses

Boron isotopes in planktonic foraminifera have been used extensively to reconstruct past ocean pH and thus CO_2_ concentrations e.g., refs. ^4,10,84,85^. Here we use the Thermo Scientific Neptune multicollector ICP-MS at the University of Southampton. External reproducibility of δ^11^B analyses is calculated from the long-term precision of consistency standards, and two relationships depending on the amplifiers used for the Faraday cups;

2 $${\mathrm{For}}\;10^{12}\,{\mathrm{amplifiers:}}\;129600 *e^{\left( { - 212 \ast ^{11}B\left( {Volts} \right)} \right)} + 0.339 * e^{\left( { - 1.544 \ast ^{11}B\left( {Volts} \right)} \right)}$$

3 $${\mathrm{For}}\;10^{11}\, {\mathrm{amplifiers:}}\;2.251 * e^{\left( { - 23.01 \ast ^{11}B\left( {Volts} \right)} \right)} + 0.278 * e^{\left( { - 0.639 \ast ^{11}B\left( {Volts} \right)} \right)}$$

The seawater boron isotopic composition (δ^11^B_sw_) for the Eocene has been estimated in ref. ^4^ based on two scenarios, one involving no vital-effect corrections (38.2–38.7‰) and one using the modern surface dwelling *Trilobatus sacculifer*^19^ δ^11^B calibration (38.6–38.9‰).

For the targeted Eocene planktonic foraminiferal species, δ^11^B vital effects as observed in modern (extant) species are likely not applicable^4^. If vital effects are present in Eocene foraminiferal δ^11^B, these only played a minor role^4,17^, supported by the demonstration that during periods of reduced δ^11^B_sw_, vital effect corrections on δ^11^B are also reduced^14^, especially for when targeting small size fraction foraminifera as in this study (Supplementary Data 1). Nonetheless, we also apply the modern *T. sacculifer* calibration^19^ (for the 300 to 355 μm size fraction), adjusting the intercept of the calibration to Eocene-specific δ^11^B_sw_ as described in ref. ^14^ (*T. sacculifer* δ^11^B-pH proxy intercept = 1.748 for average δ^11^B_sw_ = 38.75‰).

This provides an upper limit on potential δ^11^B vital effects in the Eocene planktonic foraminifera selected here. Notably, our calculated pH and CO_2_ estimates for both approaches are largely within uncertainty (Supplementary Data 1).

### Second carbonate parameter

After computing seawater pH using Eocene δ^11^B_sw_ and foraminiferal δ^11^B, an additional carbonate parameter is required to calculate CO_2_ concentrations at any given seawater salinity and temperature. Here, the second parameter we use is the surface oceanic saturation of calcite (surface Ω_calc_ = [Ca]_sw_ ∗ [CO_3_^2−^]/K_sp_), estimated at different paleolatitudes^4^. For IODP 1407/1409, Ω_calc_ is estimated at 4.5 ± 1, for ODP 865 and ODP 1258/1260 Ω_calc_ is estimated at 6.5 ± 1, for the re-processing of the δ^11^B data of^10^ from DSDP 401 we used Ω_calc_ = 5.5 ± 1, and for the data from ODP1209/1210 and ODP 1265 in ref. ^9^ we used Ω_calc_ = 6 and 4.5 (±1), respectively. In support of the narrow range of potential Ω_calc_, a variety of carbon cycle modelling studies of the early Cenozoic oceans show that surface water Ω_calc_ remains, within ±1, essentially constant and independent of model boundary conditions^16,85,86^.

### Monte Carlo pH-CO_2_ estimates from planktonic foraminiferal δ^11^B

We followed established methods to calculate seawater pH and CO_2_ from foraminiferal δ^11^B^12–14^. Atmospheric CO_2_ was calculated using a Monte Carlo approach to solve the relevant carbonate system equations with 1000 iterations, deriving mean, upper and lower bounds of 95% of the simulations. We use the seawater Ca and Mg concentrations and salinity constraints in ref. ^4^ and the equation in ref. ^12,13^ to correct for ion pairing. For each CO_2_ estimate, the Mg/Ca derived temperature from the same aliquot was used, with a ±2 °C uncertainty. All simulations were iterative assuming Gaussian distribution of these parameters within the stated 2 sigma error envelope of the mean. Note that a Gaussian distribution is not applicable to δ^11^B_sw_ because there is equal likelihood that it lay between the minimum and maximum constraints; we therefore applied a uniform probability δ^11^B_sw_ for the Monte Carlo simulations.

### GMT calculations

We convert the ODP 959 TEX_86_ SST record of ref. ^1^ to GMT, employing previously published model simulations with the NCAR CESM version 1 with CAM 4^1^, which essentially provides a transfer function from SST at ODP 959 to a global mean in four specific time windows (54–49, 48–46, 42–42, 38–35 Ma; Supplementary Fig. 2). The regression is then:

4 $${\mathrm{GMT}} = 0.91( {\pm 0.04}) \times {\mathrm{SST}}\;\left( {{\mathrm{ODP}} \, 959,\;{\mathrm{TEX}}_{86}} \right)-6.66\;(\pm 1.3)\;(1{\mathrm{s.d.}})$$

Previous model simulations of ocean temperature are consistent with both proxy estimations of SST and deep-sea temperatures at multiple locations^1^. It is important to note that the calculation does not depend on the climate sensitivity of the model, just the relationship between local and global temperature. The resulting relationship between GMT and SST from ODP 959 is then interpolated for the remaining part of the TEX_86_ record in ref. ^1^, resulting in a time-resolved GMT record for the Eocene (Fig. 5c). A similar GMT record is generated when the same approach is applied to the tropical SST compilation^1,2,57,65,87–93^ summarized in ref. ^1^ and Fig. 4, albeit with greater noise possibly the result of inconsistencies in tuning the transfer function for multiple sites and for different time intervals of the curve (Supplementary Figs. 2 and 5). The agreement between GMT records estimated from ODP 959 compared to the tropical-multi site compilation confirms that this approach is not dependent on the regional temperature, as long as the tie points are able to capture the major variations in each time series. The relevant uncertainty for each estimate of GMT (Fig. 5 and Supplementary Data 2) is the product of 1000 realization of TEX_86_-temperature reconstruction and analytical uncertainty^1^, randomly sampled within its 95% CI uncertainty envelope, including the standard errors of the regression (Supplementary Fig. 2).

## Supplementary information

Supplementary Information

Description of Additional Supplementary Files

Supplementary Data 1

Supplementary Data 2

Supplementary Data 3

## Data Availability

The authors declare that all data supporting the findings of this study are available within the Supplementary Information and Supplementary Data files associated with this paper.
