# Supplementary material for: Proxy evidence for state-dependence of climate sensitivity in the Eocene greenhouse
Source: Nat Commun. 2020 Sep 7;11:4436. doi: 10.1038/s41467-020-17887-x (PMC7477227; doi:10.1038/s41467-020-17887-x)
Supplement: Supplementary file 1 — Supplementary Information [file 41467_2020_17887_MOESM1_ESM.pdf]

Supplementary Information

Proxy evidence for state-dependence of climate sensitivity in the Eocene greenhouse

by Anagnostou et al.

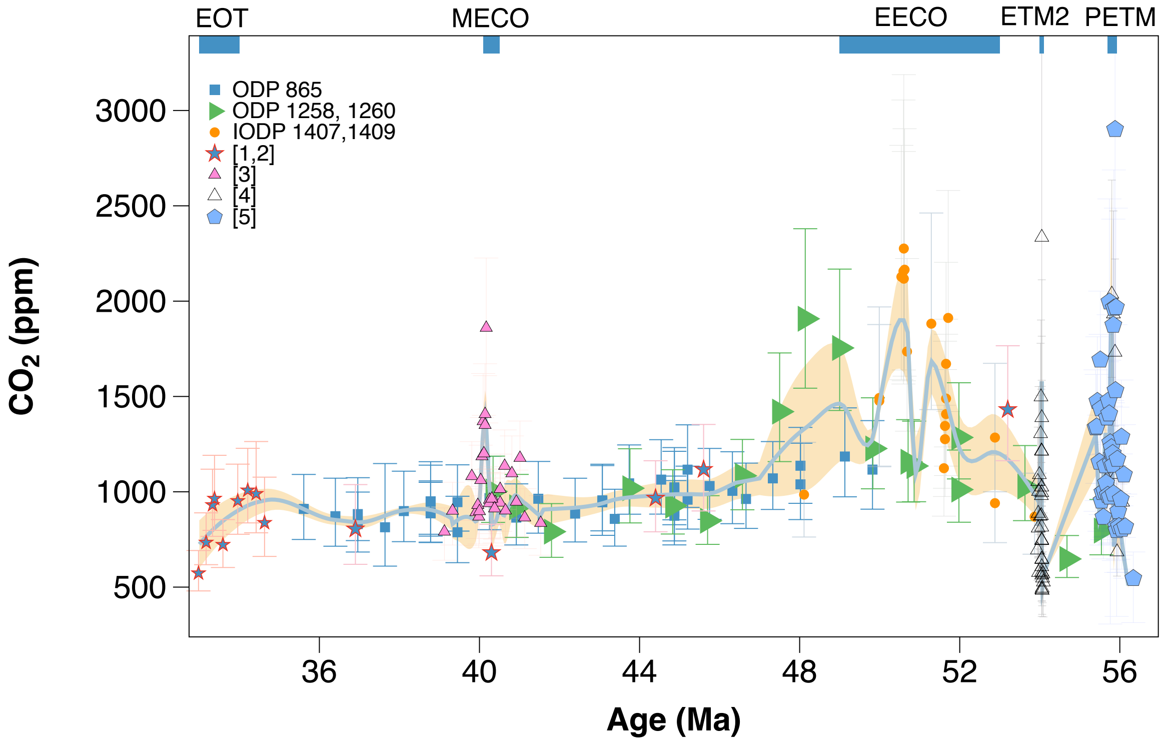

Supplementary Figure 1: Compilation of multi-site δ^{11}B-derived CO_2 for the Eocene. The Middle Eocene Climatic Optimum (MECO)[3], Eocene Thermal Maximum 2 (ETM2)[4] and Paleocene-Eocene Thermal Maximum (PETM)[4,5] records were reprocessed, as described in the Methods, to be consistent with the rest of the data in the time series. The line and shaded region represent LOESS curve and its 95% confidence. All data and errors are summarized in Supplementary Data 1and 3.

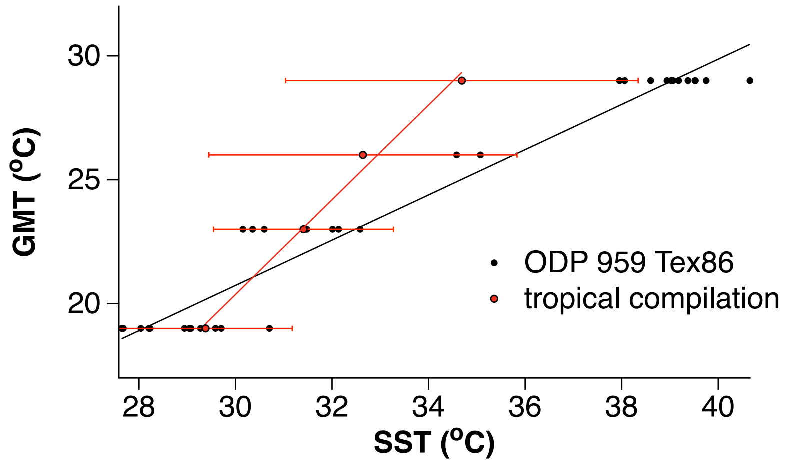

Supplementary Figure 2: Transfer functions between Global Mean Temperature (GMT) tie points and either the ODP 959 TEX_{86} record (black), or the tropical Sea Surface Temperature (SST) compilation (red). All data are summarized in Ref. [6], and also shown in Supplementary Fig. 5.

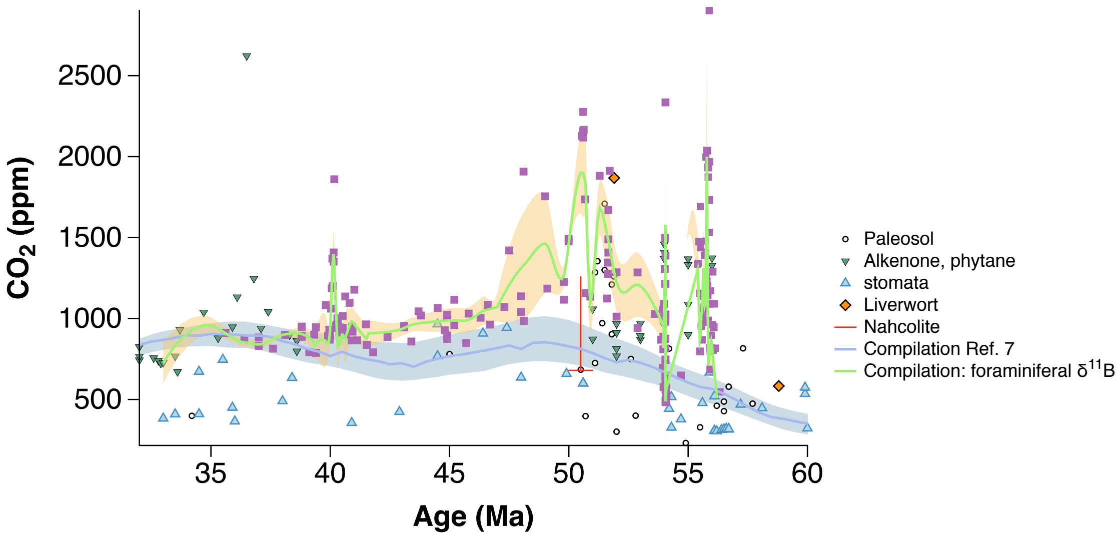

Supplementary Figure 3: Compilation of CO_2 records for the Eocene. Data are from the compilation in Ref. [7], including the new δ^{11}B-derived CO_2 in this study and in Refs. [3-5], phytane-CO_2 [8], and stomata-CO_2 [9]. Note that the Nahcolite estimates best represent a minimum in atmospheric CO_2. Lines and shaded envelopes represent LOESS curves and their 95% confidence for the compilation in Ref. [7] (purple and blue) and the compilation from foraminiferal δ^{11}B in this study (green and orange).

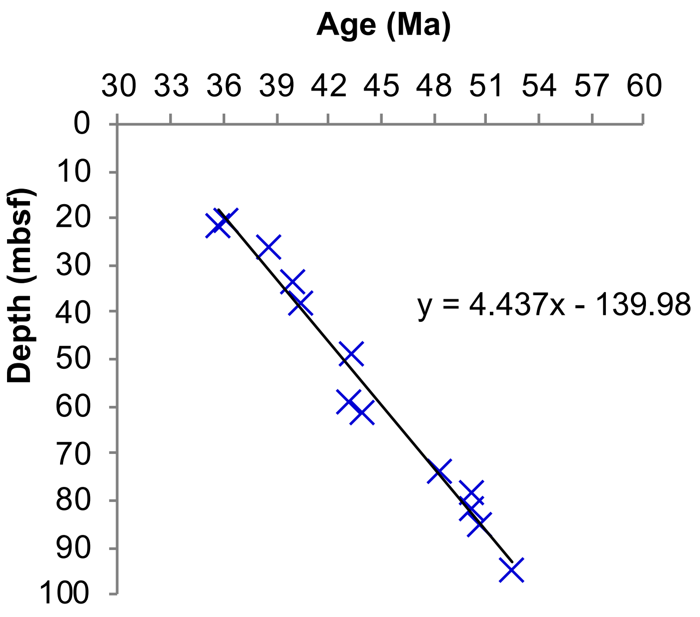

Supplementary Figure 4: Age model used for ODP Site 865B in this study. The model is based on a linear fit through planktonic foraminiferal datums of Ref. [10] as modified here (Supplementary Table 1).

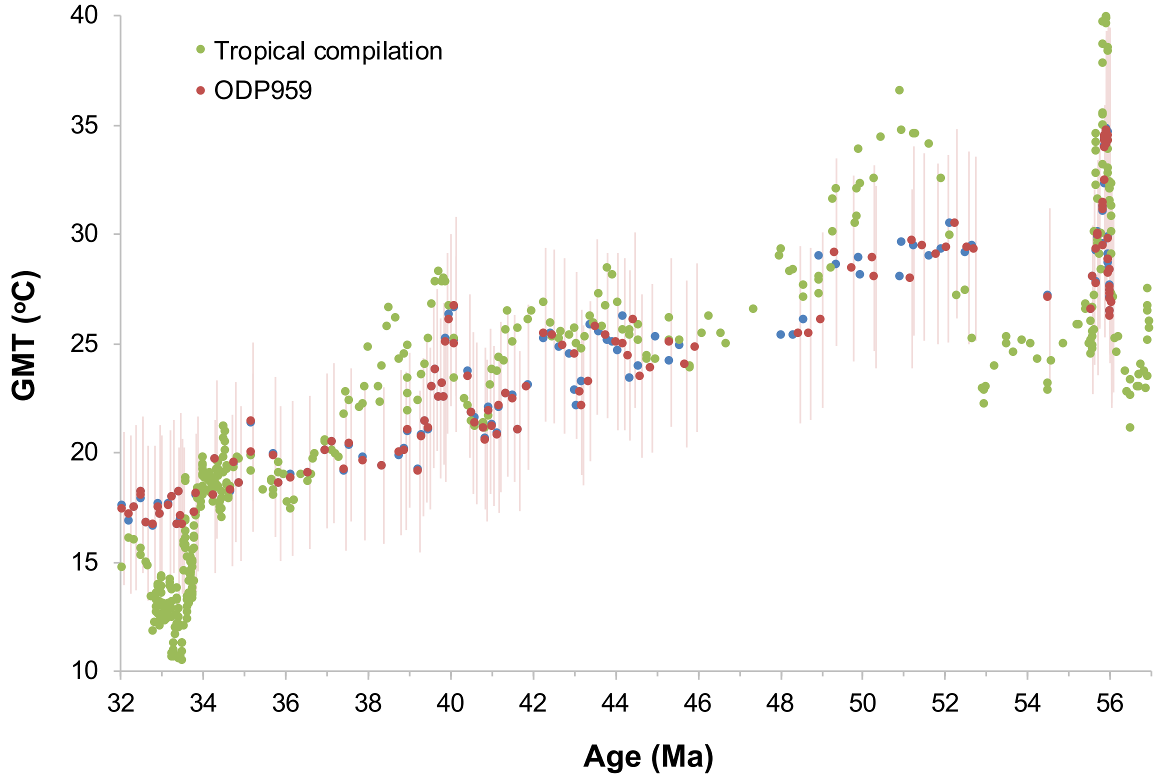

Supplementary Figure 5: Derived GMT time series for the Eocene. Brown symbols are based on the ODP 959 TEX_{86} record[6]. The green circles are based on the tropical compilation of Ref. [6], which includes Ref. [6,11-20]. Brown symbols are on the Ref. [21] age model. Blue symbols are the same as the brown but on the GTS2012 [22] age model, demonstrating the magnitude of potential misalignment due to the age model chosen. Error bars represent 1 s.d. uncertainties.

Planktonic foraminifera datum	Mean datum depth (mbsf)	Age (Ma) GTS2012 (± 0.169)
Top *Globigerinatheka semiinvoluta* [10]	20.35	36.18
Base *Turborotalia cunialensis*	21.81	35.71
Base *G. semiinvoluta*	26.3	38.62
Top *Orbulinoides beckmanni* [10]	33.63	40.03
Base *O. beckmanni* [10]	37.91	40.49
Top *Morozovella aragonensis*	48.88	43.26
Base *M. lehneri*	59.25	43.15
Base *G. kugleri*	61.28	43.88
Base *T. frontosa* [10]	73.94	48.31
Base *Astrorotalia palmerae* [10]	78.15	50.2
Base *Acarinina cuneicamerata*	81.81	50.2
Top *M. subbotinae*	85.35	50.67
Base *M. aragonensis*	94.85	52.54

Supplementary Table 1: Age model for ODP Site 865B. Planktonic foraminiferal datums, mean datum core depths, and GTS2012 datum ages.

Supplementary References

1. Anagnostou, E. *et al.* Changing atmospheric CO_2 concentration was the primary driver of early Cenozoic climate. *Nature* **533**, 380-384 (2016).
2. Pearson, P. N., Foster, G. L. & Wade, B. S. Atmospheric carbon dioxide through the Eocene-Oligocene climate transition. *Nature* **461**, 1110-1113 (2009).
3. Henehan, M.J. *et al.* Revisiting the Middle Eocene Climatic Optimum 'Carbon Cycle Conundrum' with new estimates of atmospheric pCO_2 from boron isotopes. *Paleoceanogr. Paleoclimatol.* **35**, e2019PA003713 (2020).
4. Harper, D. T. *et al.* The magnitude of surface ocean acidification and carbon release during Eocene Thermal Maximum 2 (ETM-2) and the Paleocene–Eocene Thermal Maximum (PETM). *Paleoceanogr. Paleoclimatol.* **35**, e2019PA003699 (2020).
5. Gutjahr, M. *et al.* Very large release of mostly volcanic carbon during the Paleocene-Eocene Thermal Maximum Paleocene-Eocene Thermal Maximum. *Nature* **548**, 573-577 (2017).
6. Cramwinckel, M. J. *et al.* Synchronous tropical and polar temperature evolution in the Eocene. *Nature* **559**, 382-386 (2018).
7. Foster, G. L., Royer, D. L. & Lunt, D. J. Future climate forcing potentially without precedent in the last 420 million years. *Nat. Commun.* **8**, 14845 (2017).
8. Witkowski, C. R. *et al.* Molecular fossils from phytoplankton reveal secular Pco_2 trend over the Phanerozoic. *Sci. Adv.* **4**, eaat4556 (2018).
9. Wolfe, A. P. *et al.* Middle Eocene CO_2 and climate reconstructed from the sediment fill of a subarctic kimberlite maar. *Geology* **45**, 619-622 (2017).
10. Coxall, H. K. *Hantkeninid Planktonic Foraminifera And Eocene Palaeoceanographic Change.* PhD thesis (University of Bristol, Bristol, 2000).
11. Evans, D. *et al.* Eocene greenhouse climate revealed by coupled clumped isotope-Mg/Ca thermometry. *Proc. Natl. Acad. Sci.* **115**, 1174 (2018).
12. Pearson, P. N. *et al.* Stable warm tropical climate through the Eocene Epoch. *Geology* **35**, 211-214 (2007).
13. Liu, Z. *et al.* Global cooling during the Eocene-Oligocene climate Transition. *Science* **323**, 1187-1190 (2009).
14. Zhang, Y. G., Pagani, M., Liu, Z., Bohaty, S. M. & DeConto, R. A 40-million-year history of atmospheric CO_2. *Phil Trans Royal Soc A* **371**, 20130096 (2013).

15. Pearson, P. N. & Burgess, C. E. Foraminifer test preservation and diagenesis: Comparison of high latitude Eocene sites. *Special Publications* **303**, 59-72 (The Geological Society, London, 2008).
16. Lear, C. H., Bailey, T. R., Pearson, P. N., Coxall, H. K. & Rosenthal, Y. Cooling and ice growth across the Eocene-Oligocene transition. *Geology* **36**, 251-254 (2008).
17. Inglis, G. N. *et al.* Descent towards the Icehouse: Eocene sea surface cooling inferred from GDGT distributions. *Paleoceanography* **30**, 1000–1020 (2015).
18. Tripati, A. K. *et al.* Tropical sea-surface temperature reconstruction for the early Paleogene using Mg/Ca ratios of planktonic foraminifera. *Paleoceanography* **18**, 1101 (2003).
19. Frieling, J. *et al.* Extreme warmth and heat-stressed plankton in the tropics during the Paleocene-Eocene Thermal Maximum. *Sci. Adv.* **3**, e1600891 (2017).
20. Frieling, J. *et al.* Tropical Atlantic climate and ecosystem regime shifts during the Paleocene–Eocene Thermal Maximum. *Clim. Past* **14**, 39-55 (2018).
21. Westerhold, T. *et al.* Astronomical calibration of the Ypresian timescale: implications for seafloor spreading rates and the chaotic behavior of the solar system? *Clim. Past* **13**, 1129-1152 (2017).
22. Gradstein, F. M. *et al.* *The Geologic Time Scale*, (Boston, USA, Elsevier, 2012).
