# Supplementary material for: Proxy evidence for state-dependence of climate sensitivity in the Eocene greenhouse
Source: Nat Commun. 2020 Sep 7;11:4436. doi: 10.1038/s41467-020-17887-x (PMC7477227; doi:10.1038/s41467-020-17887-x)
Supplement: Supplementary file 2 — Description of Additional Supplementary Files [file 41467_2020_17887_MOESM2_ESM.pdf]

Description of Additional Supplementary Files

File Name: Supplementary Data 1
Description: Site core information, foraminifera samples, analyses, and calculations.

File Name: Supplementary Data 2
Description: Global Mean Temperature (GMT) estimates, CO_2 forcing relative to preindustrial, and climate sensitivity calculations.

File Name: Supplementary Data 3.
Description: Reprocessed $\delta^{11}B$ data for atmospheric CO_2 and seawater pH from planktonic foraminifera. Note that for this processing, no vital effect corrections are applied, and temperature is based on Mg/Ca or $\delta^{18}O$ measurements in the respective references, treated the same way as for the rest of the time series in this paper.
